# The HD-ZIP II Transcription Factors Regulate Plant Architecture through the Auxin Pathway

**DOI:** 10.3390/ijms21093250

**Published:** 2020-05-04

**Authors:** Guanhua He, Pan Liu, Huixian Zhao, Jiaqiang Sun

**Affiliations:** 1 College of Life Sciences, Northwest A & F University, Yangling, Shaanxi 712100, China; hghqwe1024@sina.cn; 2 National Key Facility for Crop Gene Resources and Genetic Improvement, Institute of Crop Sciences, Chinese Academy of Agricultural Sciences, Beijing 100081, China; liupan503@163.com

**Keywords:** *Arabidopsis*, wheat, HD-ZIP, ATHB2, auxin

## Abstract

The homeodomain-leucine zipper (HD-ZIP) family transcription factors play important roles in plant growth and development. However, the underlying mechanisms remain largely unclear. Here we found that *ATHB2*, encoding a HD-ZIP transcription factor, is an early auxin responsive gene. Phenotypic analyses show that overexpression of *ATHB2* impairs plant architecture, including reduced plant height and small leaves, and also reduces auxin response in leaves when grown in soil. Simultaneously, the seedlings with chemical induction of *ATHB2* exhibit abnormal root gravitropism, a typical auxin-related phenotype. We further show that the auxin response pattern is altered in roots of the inducible *ATHB2* seedlings. Consistently, the transcript levels of some auxin biosynthetic and transport genes are significantly decreased in these transgenic seedlings. Further, protein and promoter sequence analyses in common wheat showed that the HD-ZIP II subfamily transcription factors have highly conserved motifs and most of these encoding gene promoters contain the canonical auxin-responsive elements. Expression analyses confirm that some of these *HD-ZIP* II genes are indeed regulated by auxin in wheat. Together, our results suggest that the HD-ZIP II subfamily transcription factors regulate plant development possibly through the auxin pathway in plants.

## 1. Introduction

The plant hormone auxin is important for regulating almost all aspects of plant growth and development [1]. Auxin receptors perceive the auxin concentration changes and then initiate auxin signaling. Nuclear auxin signal perception and consequent alterations in gene expression are carried out by the TRANSPORT INHIBITOR RESPONSE1/AUXIN SIGNALING F-BOX (TIR1/AFB) pathway [2,3]. Three major protein families are involved in the TIR1/AFB pathway: auxin-binding TIR1/AFB F-box proteins, AUXIN/INDOLE-3-ACETIC ACID INDUCIBLE (Aux/IAA) repressor proteins, and AUXIN RESPONSE FACTOR (ARF) transcription factors [3,4]. In this pathway, the Aux/IAAs interact with ARFs and inhibit ARF transcription factor activity when the auxin level is low [5]. When the auxin level is increased, the Aux/IAA and TIR1/AFB F-box proteins form a co-receptor complex, which are components of a Skp1-Cullin-F-box (SCF) E3 ubiquitin ligase complex, resulting in ubiquitination and degradation of the Aux/IAA repressor proteins by the 26S proteasome [1,5,6]. Recently, Cao et al. (2019) identified another auxin signaling mechanism, which acts in parallel to the canonical auxin pathway [7]. This signaling mechanism works at the concave side of the apical hook. The auxin-mediated C-terminal cleavage of transmembrane kinase 1 (TMK1) is involved in this signaling [7].

Two distinct pathways are involved in auxin distribution in the plant body: passive diffusion through the plasma membrane (PM) and directional cell-to-cell polar auxin transport (PAT) [8]. PAT is a precise auxin distribution manner that is significantly important for the formation of local auxin maxima, mainly in developing tissues [8,9]. The differential localization of transporters at specific sites on the PM creates a directional auxin flow, which eventually establishes a PAT stream through adjacent cells [9]. AUXIN RESISTANT1 (AUX1)/LIKE-AUX1 (LAX) proteins are auxin influx transporters and PIN-FORMED (PIN) proteins are auxin efflux transporters [10,11,12,13,14,15]. Other auxin transports, which have nonpolar distribution, consist of the P-GLYCOPROTEIN (PGP), MULTIDRUG RESISTANCE (MDR), or ATP-BINDING CASSETTE SUBFAMILY B (ABCB). AUX/LAX regulates several developmental processes, such as lateral root formation (AUX1 and LAX3) and cotyledon vascular patterning (LAX2) [15,16]. Among the distinct auxin transports, the polar localizations of PIN proteins within cells finely correspond to the directionality of auxin flow, which highlights that PIN proteins that are mainly responsible for the asymmetric distributions of auxin in plants [16,17,18]. The homeodomain-leucine zipper (HD-ZIP) transcription factors are unique to plants and contribute to various plant physiological processes [19,20,21,22]. Based on evolutionary relationship and protein structure, HD-ZIP transcription factors are generally divided into four subfamilies, namely HD-ZIP I, HD-ZIP II, HD-ZIP III and HD-ZIP IV [23]. HD-ZIP II proteins can be stimulated by far red light, and then lead to shade avoidance response [24,25]. Previous studies showed that HD-ZIP II proteins respond to various biotic and abiotic stresses, through regulating hormone signaling pathways and expression of related genes [26,27,28]. A recent study showed that HD-ZIP II proteins regulate shoot apical meristems (SAM) maintenance and cotyledon polarity through auxin flow, but there is no evidence to suggest how HD-ZIP II proteins regulate auxin perception or auxin response [29,30].

In this study, we identified an early auxin responsive gene, *ATHB2,* which is one of the HD-ZIP II subfamily proteins in *Arabidopsis.* Phenotypic observation showed that overexpression of *ATHB2* impaired plant architecture, including reduced plant height and small leaves and decreased auxin response in leaves when grown in soil. Meanwhile, *ATHB2*-inducible expression led to a typical auxin-related phenotype known as the agravitropic root phenotype. Furthermore, confocal microscopic analyses showed that asymmetric auxin response occurred in roots of *ATHB2*-overexpressing seedlings. We also found that the expression levels of some auxin biosynthetic and transport genes were reduced in the *iATHB2* seedlings. Further, we found that most of the *HD-ZIP* II subfamily gene promoters in common wheat contain the canonical auxin-responsive elements. Expression analyses confirm that some of these *HD-ZIP* II genes are indeed regulated by auxin in wheat. Together, our results suggest that the HD-ZIP II subfamily transcription factors regulate plant development possibly through the auxin pathway in plants.

## 2. Results

### 2.1. ATHB2 Is an Early Auxin-Inducible Gene

**Figure 1:**
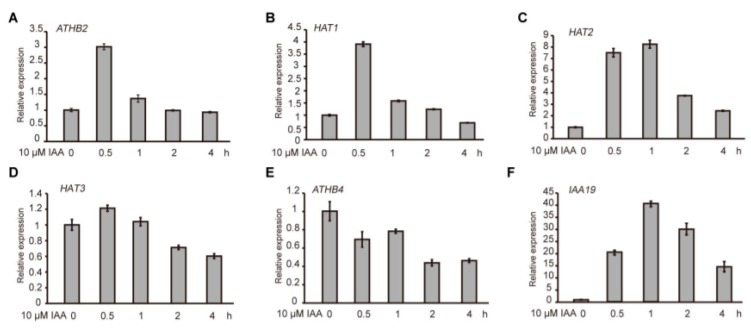
Expression patterns of the *ATHB2* subset genes. (**A**–**F**) Expression analyses of *ATHB2, HAT1, HAT2, HAT3, ATHB4* and *1AA19* in response to auxin treatment. The 6 day old wild-type (Col-0) seedlings were treated with 10 μM IAA for different time points. The *ACTIN7* gene was used as an internal reference. The qRT-PCR results were performed for three biological replications and similar results were observed. Representative qRT-PCR results with three technical replicates were shown. Error bars denote ± SD.

ATHB2 belongs to a member of the HD-ZIP II subfamily transcription factors in *Arabidopsis*. A previous study showed that the three proteins *ATHB2*, *HAT3* and *ATHB4* redundantly regulate embryo development in plants [30]. However, the underlying mechanism remains largely unknown. To investigate whether the biological role of ATHB2 and its homologs (Figure S1) is related to the auxin pathway, the 6 day old wild-type (Col-0) seedlings were treated with 10 μM IAA for different time points. The quantitative reverse transcription polymerase chain reaction (qRT-PCR) results showed that the transcript levels of the *ATHB2* gene increased to a maximum level about three-fold at 0.5 h after exogenous IAA treatment (Figure 1A). The auxin-mediated induction of *ATHB2* expression was substantially reduced after 1 h of treatment (Figure 1A). Similarly, the transcript levels of *HAT1* and *HAT2* genes were also obviously elevated after IAA treatment for 0.5 h (Figure 1B,C). By contrast, the transcriptional expression of *HAT3* and *AHTB4* was downregulated by IAA treatment (Figure 1D,E). The *Aux/IAA* genes are well known as the early auxin responsive genes and participate in auxin signaling through interacting with ARFs as transcriptional repressors. As a control, the transcriptional expression of *IAA19,* one representative member of the *Aux/IAA* family, was rapidly increased at 0.5 h after exogenous IAA treatment and reached a maximum at 1 h (Figure 1F). Taken together, like *Aux/IAA* genes, *ATHB2* and its close homologs *HAT1* and *HAT2* are early auxin responsive genes.

### 2.2. Overexpression of ATHB2 Affected Plant Architecture

**Figure 2:**
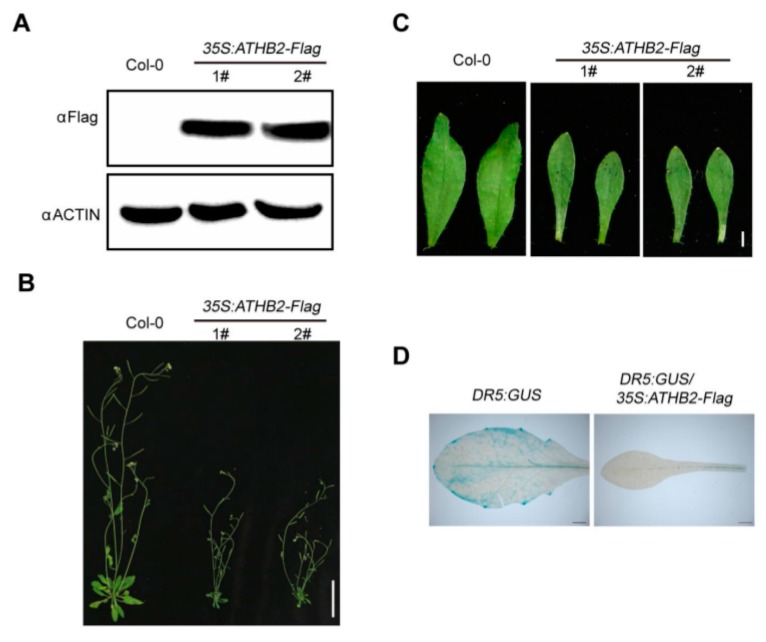
Morphological phenotype of *35S:ATHB2-FLAG* transgenic lines. (**A**) Immunoblotting analysis showing the ATHB2-FLAG protein levels in the *35S:ATHB2-FLAG* transgenic plants. The 6 day old seedlings of Col-0 and *35S:ATHB2-FLAG* transgenic plants were harvested for immunoblotting analysis. ACTIN was used as a loading control. 1# and 2# represent two independent transgenic lines of *35S:ATHB2-FLAG.* The data are representative of three independent experiments. (**B** and **C**) Overview of the Col-0 and *35S:ATHB2-FLAG* transgenic lines at the adult stage. (**B**) Scale bars, 4cm. Leaf morphology of the Col-0 and *35S:ATHB2-FLAG* transgenic lines at the adult stage. (**C**) Scale bars, 2 mm. The 4-week-old *35S:ATHB2-FLAG* transgenic plants grown under normal growth conditions were used for phenotypic analyses. (**B** and **C**) Two independent transgenic lines were used for phenotype observation. The images are representative of three independent experiments. (**D**) Expression patterns of *DR5:GUS* in the leaves of *DR5:GUS* and *DR5:GUS/35S:ATHB2-FLAG* plants. The leaves of 3-week-old *DR5:GUS* and *DR5:GUS/35S:ATHB2-FLAG* plants were used for GUS activity analyses. The images are representative of three independent experiments.

To observe the morphological phenotypes of *ATHB2* overexpression plants at the adult stage, we generated different *35S:ATHB2-FLAG* transgenic lines constitutively overexpressing *ATHB2*. Western blotting analyses demonstrated that the ATHB2-FLAG fusion proteins accumulated in the *35S:ATHB2-FLAG* transgenic seedlings (Figure 2A). Phenotypic analyses showed that the 4-week-old *ATHB2* overexpressing plants under normal growth conditions exhibited auxin-related phenotypes, such as dwarfism and narrow leaf phenotypes (Figure 2B,C). To explore how ATHB2 interacts with auxin to differentially regulate leaf development in the Col-0 and *35S:ATHB2-FLAG* transgenic plants, we examined the spatial distribution of the auxin response in Col-0 and *35S:ATHB2-FLAG* transgenic plants using the auxin-responsive reporter *DR5:GUS* [31]. The double transgenic plant *DR5:GUS/35S:ATHB2-FLAG* was generated through genetic crossing between *DR5:GUS* and *35S:ATHB2-FLAG* plants. *DR5:GUS* and *DR5:GUS/35S:ATHB2-FLAG* plants were used for this experiment. As shown in Figure 2D, the expression of *DR5:GUS* in the 4-week-old leaves of *DR5:GUS/35S:ATHB2-FLAG* plants was significantly decreased compared with that in the wild type (Figure 2D). These results showed that constitutive overexpression of *ATHB2* reduced auxin response and affected leaf development.

### 2.3. Inducible-Expression of ATHB2 Led to Auxin-Related Root Phenotypes

**Figure 3:**
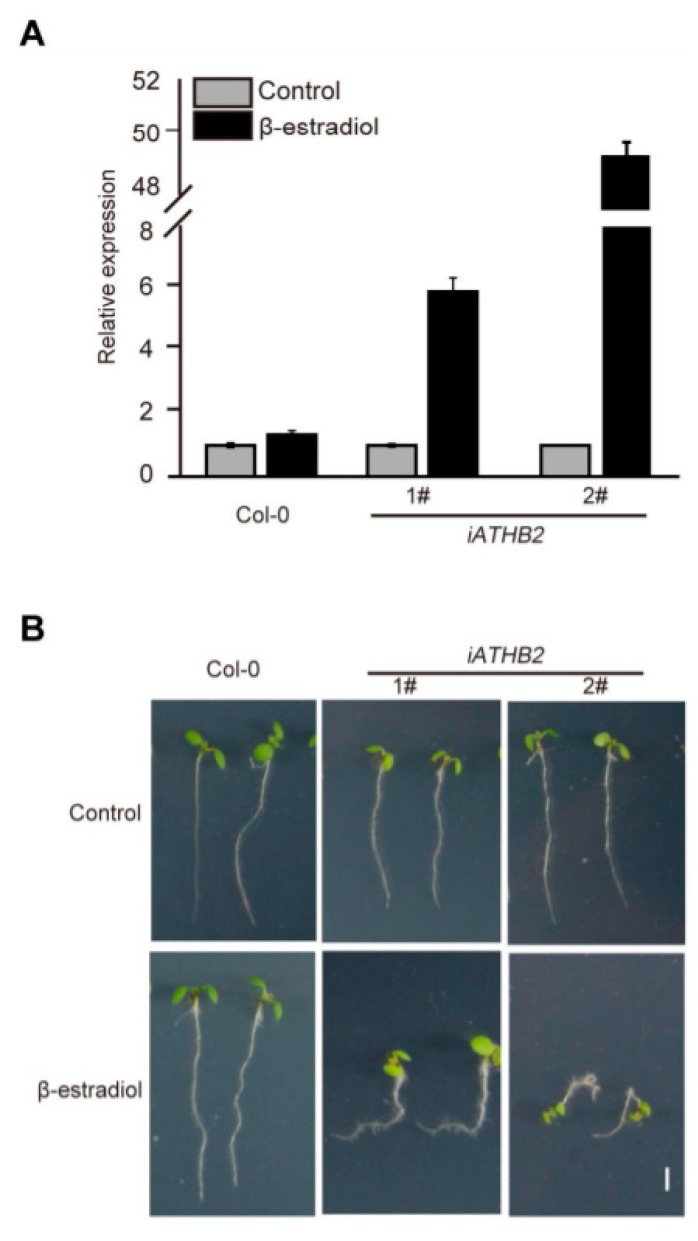
Root gravitropic phenotype of β-estradiol-inducible ATHB2-overexpression transgenic lines. (**A**) qRT-PCR analysis showing the β-estradiol-induced expression pattern of *ATHB2* in the *iATHB2* lines. The *ACTIN* gene was used as an internal reference. 1# and 2# represent two independent transgenic lines of *iATHB2.* The qRT-PCR results were performed for three biological replications and similar results were observed. Representative qRT-PCR results with three technical replicates were shown. Error bars denote ± SD. (**B**) Root gravitropic phenotypes of the *iATHB2* transgenic lines. Seedlings of the Col-0 and inducible *ATHB2* overexpression plants (*iATHB2*) were grown on 1/2 MS medium with or without 10 μM β-estradiol for six days. Two independent transgenic lines were used for phenotypic observation. The images are representative of three independent experiments.

In order to determine the biological relevance of ATHB2 in auxin-mediated physiological processes, the β-estradiol-inducible *ATHB2* overexpressing plants (XVE>>ATHB2*,* simply labeled as *iATHB2*) were generated. The qRT-PCR results showed that the transcript levels of *ATHB2* were significantly increased in the inducible transgenic lines after β-estradiol treatment for 2 h compared with the Col-0 seedlings (Figure 3A). Notably, the transcript levels of *ATHB2* were greatly upregulated by about 60-fold in the *iATHB2* 2# transgenic line (Figure 3A).

To observe the auxin-related phenotype of *iATHB2* transgenic lines, the 6-day-old *iATHB2* seedlings were grown on 1/2 Murashige and Skoog (MS) medium with or without 10 μM β-estradiol. In the absence of β-estradiol, the growth phenotype of *iATHB2* and Col-0 seedlings was comparable. However, two independent *iATHB2* transgenic lines displayed an agravitropic root phenotype grown on the medium containing 10 μM β-estradiol, whereas the root growth of Col-0 plants was not affected in the presence of 10 μM β-estradiol (Figure 3B). In addition, the primary root length analyses were performed using Col-0 and *iATHB2* transgenic seedlings grown on the medium containing 10 μM β-estradiol. As shown in Figure S2, the primary root length of *iATHB2* transgenic seedlings was significantly reduced compared with that of Col-0 seedlings. These results suggest that ATHB2 might be involved in the auxin-mediated root development.

### 2.4. Inducible-Expression of ATHB2 Altered Auxin Distribution in Roots

**Figure 4:**
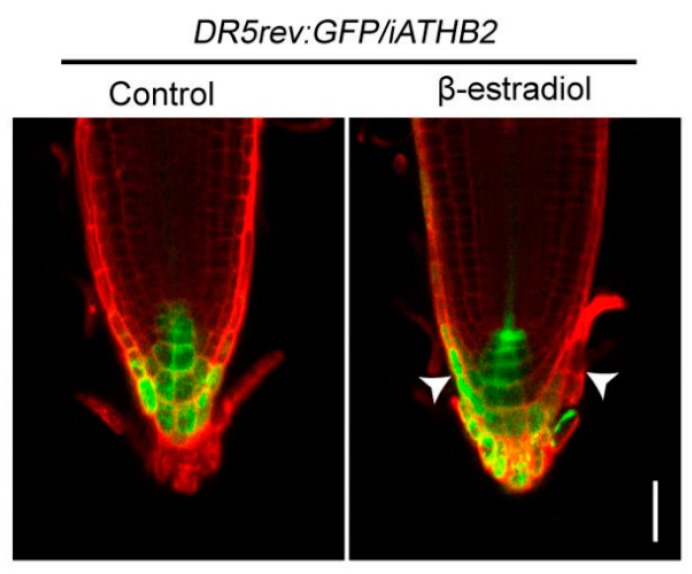
Asymmetric auxin response was observed in the root tips of *iATHB2* plants. The spatial expression pattern of the *DR5rev:GFP* auxin responsive reporter in the root tips of 6-day-old *DR5rev:GFP/iATHB2* plants grown on the medium with or without 10 μM β-estradiol. The images are representative of three independent experiments. Roots were stained with propidium iodide (red). Arrowheads indicate the asymmetric auxin response in the root tips. Scale bars = 50 μm. Arrowheads indicate the asymmetric auxin response of *DR5rev:GFP/iATHB2* with 10 μM β-estradiol treatment.

Considering that root gravitropic bending is triggered by the asymmetric auxin distribution in the root tip [32,33], we examined the spatial expression pattern of the auxin responsive reporter *DR5rev:green fluorescent protein* (*GFP*) [34] in the root tips of *iATHB2* seedings. We here generated the double transgenic plant *DR5rev:GFP/iATHB2* through genetic crossing between *DR5rev:GFP* and *iATHB2* plants. After growing on 1/2 MS medium supplemented with or without 10 μM β-estradiol for six days, the fluorescence signals were observed in the root tips of *DR5rev:GFP/iATHB2* plants. In the presence of 10 μM β-estradiol, the fluorescence signals of *DR5rev:GFP/iATHB2* were asymmetric distribution in root tips as shown in the representative image (15/18) (Figure 4). However, the asymmetric *DR5rev:GFP* fluorescence signal pattern was not observed in the root tips of *DR5rev:GFP/iATHB2* plants in the absence of 10 μM β-estradiol (Figure 4). These findings indicate that the asymmetric auxin response in the root tips of *iATHB2* seedlings with β-estradiol treatment is correlated with their agravitropic root phenotypes.

### 2.5. Expression of Some Auxin Biosynthetic and Transport Genes Was Reduced in the iATHB2 Seedlings

**Figure 5:**
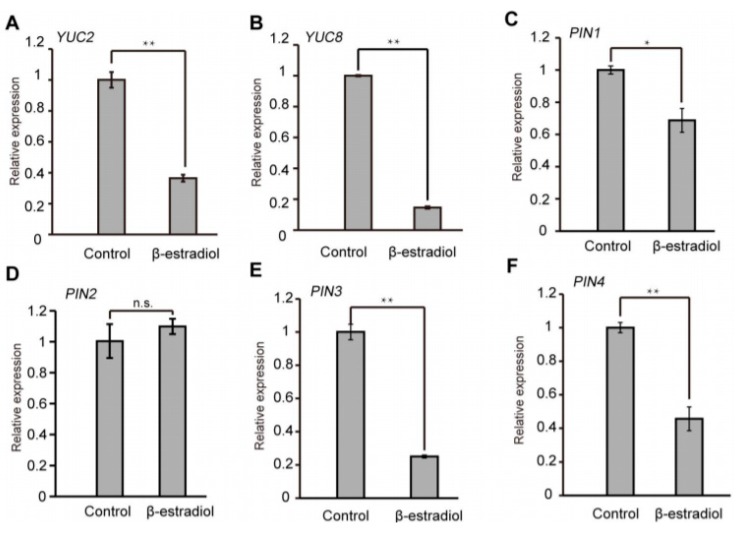
Overexpression of *ATHB2* reduced the expression levels of some auxin biosynthetic and transport genes. (**A***–***F**) Expression patterns of *YUC2*, *YUC8* and several *PINs* genes in the *iATHB2* transgenic lines. The different 6-day-old *iATHB2* transgenic lines were treated with or without 10 μM β-estradiol for 2 h. The *ACTIN7* gene was used as an internal reference. The shown data are results of the representative *iATHB2* 2# transgenic line. The qRT-PCR results were performed for three biological replications and similar results were observed. Representative qRT-PCR results with three technical replicates were shown. Error bars denote ± SD. * *p* < 0.05, ** p < 0.01, Student’s *t* test. No significant difference is shown by n.s.

Auxin levels or distribution are controlled through synthesis and transport [35]. The auxin efflux carrier PIN proteins direct auxin flow in plants [36]. To investigate the underlying mechanism of asymmetric auxin response in the root tips of *iATHB2* transgenic lines, we examined the expression of auxin biosynthetic and transport genes in the *iATHB2* transgenic lines. The *iATHB2* transgenic seedlings were grown on 1/2 MS medium for six days and then treated with 10 μM β-estradiol. As shown in Figure 5A–F, the transcript levels of *YUCCA2* (*YUC2*)*, YUC8, PIN1*, *PIN3* and *PIN4* were obviously decreased after β-estradiol treatment for 2 h. Previous studies have shown that the auxin-efflux facilitator PIN2 is involved in root gravitropism [12,36,37]. The transcriptional expression of *PIN2* has no significant change in the *iATHB2* transgenic seedlings after β-estradiol treatment. Taken together, these results indicated that the expression of some auxin biosynthetic and transport genes was reduced in the *iATHB2* seedlings after β-estradiol treatment.

### 2.6. Molecular Characterization of HD-ZIP II Proteins in Common Wheat

**Figure 6:**
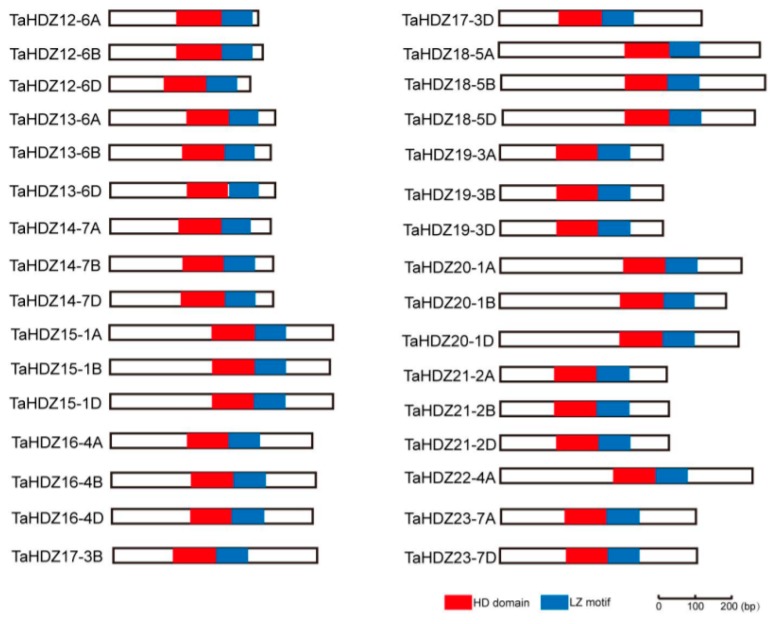
Schematic diagrams of the conserved domain structure of wheat HD-ZIP II subfamily proteins. Red box indicates the homeobox domain (HD) and blue box indicates the adjacent leucine zipper (LZ) motif, respectively.

In this study, we have shown that ATHB2, one member of the HD-ZIP II subfamily transcription factor in *Arabidopsis*, is involved in the regulation of plant architecture potentially through the auxin pathway. To further understand the roles of HD-ZIP II proteins in monocot crops, we analyzed the protein structure and motif composition of HD-ZIP II subfamily proteins in common wheat. In a recent study, a total of 32 HD-ZIP II subfamily genes were identified in common wheat [38]. Protein structure analysis was conducted via the SMART software using the 32 HD-ZIP II proteins in common wheat, indicating that all the 32 HD-ZIP II proteins contain a homeobox domain (HD) and an adjacent leucine zipper (LZ) motif (Figure 6). The evolutionary relationships among the HD-ZIP II subfamily proteins of both *Arabidopsis* and wheat were analyzed via the MEGA7.0 software using the neighbor-joining method (Figures S1 and S3).

**Figure 7:**
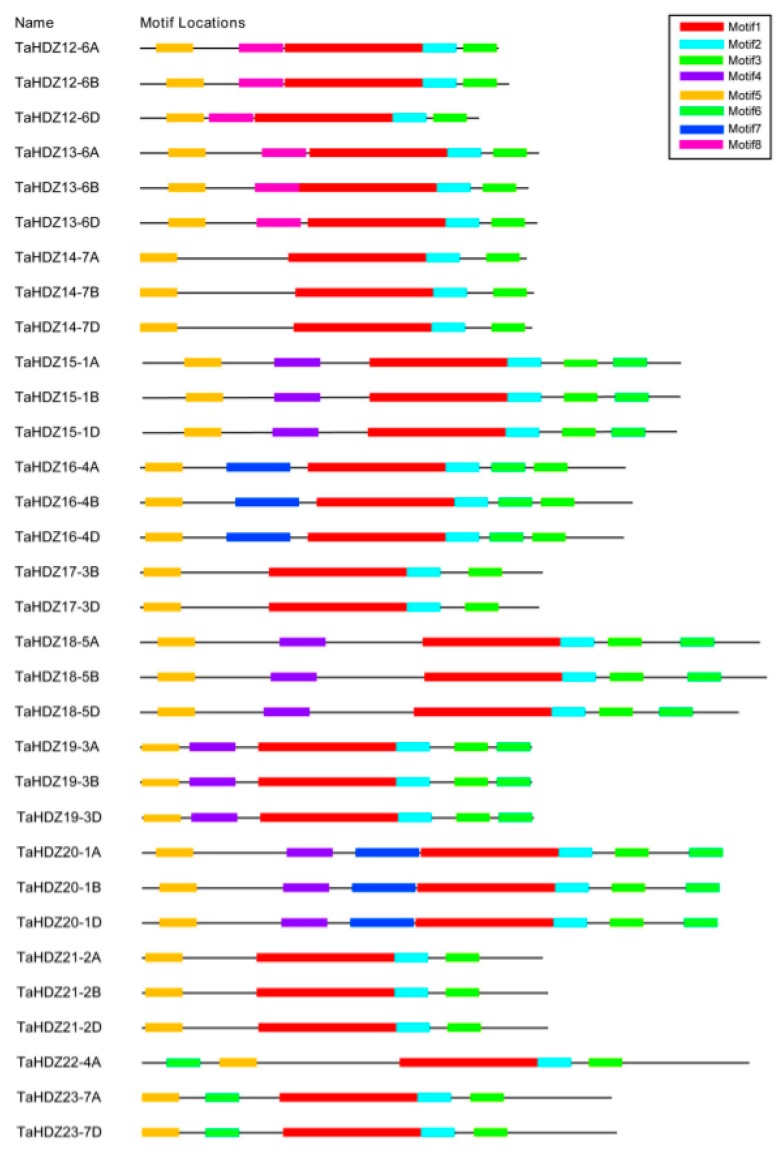
Putative motifs of the wheat HD-ZIP II subfamily proteins using the MEME program. The different conserved motifs are marked by different colors.

To comprehensively identify the potential conserved domains of wheat HD-ZIP II transcription factors, the full-length amino acid sequences were analyzed by the MEME online server. The results showed that eight predicted conserved motifs were identified, named as Motif 1 to 8 (Figure 7). As shown in Figure 7, each member of wheat HD-ZIP II subfamily proteins contains four common predicted motifs, including Motifs 1, 2, 3 and 5. Motif 1, a putative HD domain, was comprised of 78 amino acids (EDDGDGGGGARKKLRLSKEQSALLEESFKEHSTLSPKQKAALARQLGLRPRQVEVWFQNRRARTKLKQTEVDCEYLKR) (Figure S4). Motif 2, a putative LZ domain, was comprised of 19 amino acids (CCETLTEENRRLQRELAEL) (Figure S4). Motif 3 and Motif 5, whose functions remain unknown, were comprised of 19 amino acids (YYMPLPATTLTMCPSCERV) and 21 amino acids (EAEEDLGLALGLSLGAGSRPS), respectively (Figure S4). In addition to these conserved motifs contained in all the HD-ZIP II subfamily members, there are four predicted motifs which are specific to some members of the HD-ZIP II proteins.

### 2.7. Auxin-Responsive Elements in the Promoters of Wheat HD-ZIP II Genes

**Figure 8:**
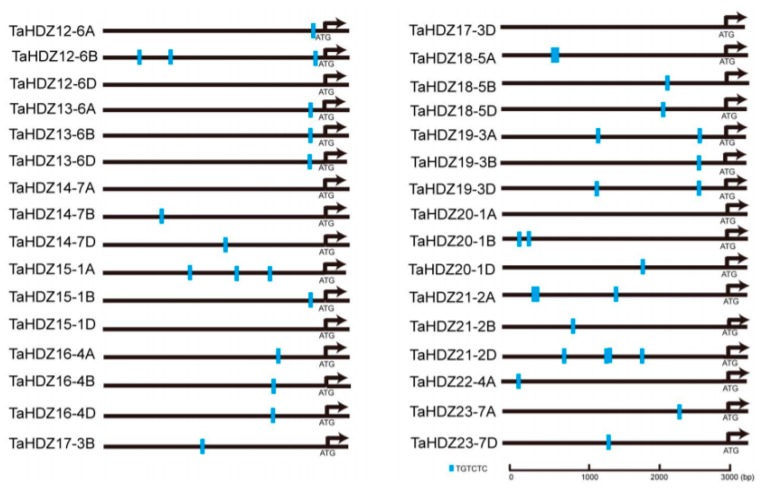
The “TGTCTC” elements in the 3 Kb genomic regions upstream of coding regions are indicated by blue box.

**Figure 9:**
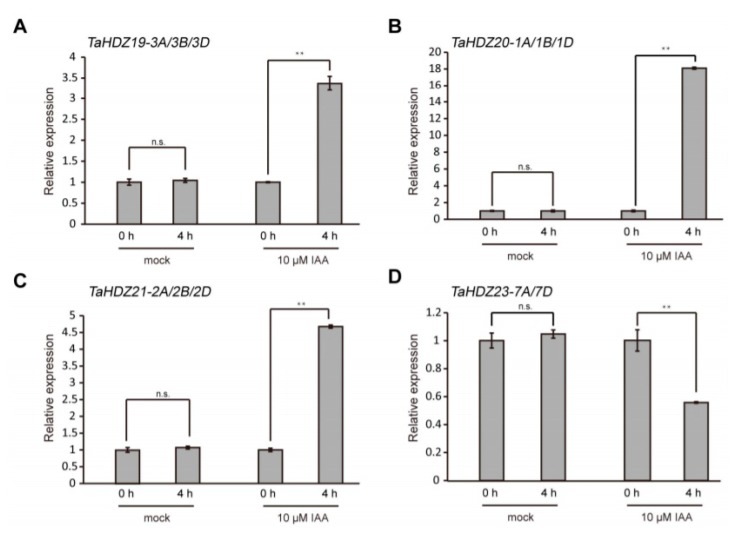
Several wheat *HD-ZIP* II subfamily genes were regulated by auxin. (**A–D**) Auxin-induced expression pattern of *TaHDZ19-3A/3B/3D, TaHDZ20-1A/1B/1D, TaHDZ21-2A/2B/2D* and *TaHDZ23-7A/7D* by qRT-PCR. The 4-day-old wheat seedlings were treated with 10 μM IAA for 4 h. The *TaGAPDH* gene was used as an internal reference. The qRT-PCR results were performed for three biological replications and similar results were observed. Representative qRT-PCR results with three technical replicates are shown. Error bars denote ± SD. ** p < 0.01, Student’s *t* test. No significant difference is shown by n.s.

Auxin-responsive promoter elements (AuxREs) presented in the upstream region of genes play an important role in the auxin pathway. The canonical AuxRE “TGTCTC” shows a strong association with the auxin responsive expression pattern [39,40]. To understand the association of wheat HD-ZIP II subfamily proteins with the auxin pathway, we scanned the “TGTCTC” element in the 3 Kb genomic regions upstream of their coding regions. Interestingly, we found that among the 32 gene promoters, only five gene promoters did not contain the canonical AuxRE “TGTCTC” (Figure 8). Notably, the *TaHDZ13-6A/6B/6D* and *TaHDZ16-4A/6B/6D* homologous genes shared almost similar “TGTCTC” distribution patterns in their promoters (Figure 8). These findings indicated that the *HD-ZIP* II subfamily genes might be regulated by auxin in wheat. To further confirm this idea, the 4-day-old wheat seedlings were treated with 10 μM IAA for 4 h for qRT-PCR analysis. The results showed that the transcript levels of *TaHDZ19-3A/3B/3D, TaHDZ20-1A/1B/1D* and *TaHDZ21-2A/2B/2D* were up-regulated in wheat roots with auxin treatment, whereas the transcript levels of *TaHDZ23-7A/7D* was downregulated (Figure 9). Taken together, some wheat *HD-ZIP* II subfamily genes are regulated by auxin.

## 3. Discussion

Auxin regulates auxin-responsive gene expression which relies on an elegantly short signal transduction pathway (TIR1/AFB pathway), which has been extensively reviewed [5,41]. The *Aux/IAA*, which are well-known as the early auxin responsive genes, act as transcriptional repressors in this signaling [31]. Aux/IAA proteins recruit corepressors of the TOPLESS (TPL) family through a conserved EAR domain to silence ARF target genes [42,43]. The Aux/IAAs do not themselves bind DNA, but they can dimerize with the ARF family transcription factors [5]. The ARF family transcription factors regulate auxin-responsive gene expression.

Previous studies have shown that several HD-ZIP II proteins are well known for their role in shade avoidance [44], carpel margin development [45] and leaf polarity [46]. Moreover, members of the HD-ZIP II subfamily also control embryonic apical patterning and SAM function [30]. In this study, we found that *ATHB2,* encoding a transcription factor of the HD-ZIP II subfamily, is an early auxin-responsive gene (Figure 1). Phenotypic analyses showed that overexpression of *ATHB2* impaired plant architecture, including reduced plant height and small leaves, which decreased auxin response in leaves when grown in soil (Figure 2). Meanwhile, the seedlings with chemical induction of *ATHB2* exhibited abnormal root gravitropism, a typical auxin-related phenotype (Figure 3). We further showed that asymmetric auxin response occurred in the root tips of inducible *ATHB2* plants (Figure 4). Therefore, both *Aux/IAA* and *ATHB2* are early auxin-responsive genes and act as repressors of the auxin pathway, but possibly through distinct mechanisms.

The asymmetrical localization of *PIN* transporters (*PIN1*–*PIN4* and *PIN7*) on PM contributes to the directionality of the auxin flow [36,47,48]. The differential expression and polar localization of PIN proteins constitutes the backbone of a transport network for directional auxin distribution in different tissues of the plant [49]. Directional auxin distribution leads to the formation of cellular auxin maxima and minima, which provides an essential cue for plant growth and differentiation at the level of individual cell and tissue [48,50]. *PIN1,* localizing to the basal (rootward) plasma membrane in root stele cells, directly transports auxin toward the root tip [12]. *PIN2*, *PIN3* and *PIN4,* which also act in the root tip, mediate the auxin maximum and auxin redistribution for root gravitropism [11,37,51]. To investigate the underlying mechanism of the *ATHB2*-regulated auxin-related phenotype, we examined the expression of some *PIN* genes in the *iATHB2* transgenic lines. qRT-PCR results showed that the transcript levels of *PIN1*, *PIN3* and *PIN4* were obviously downregulated by inducible overexpression of *ATHB2* (Figure 5). Meanwhile, the auxin biosynthetic genes *YUC2* and *YUC8* were also downregulated by *ATHB2* overexpression (Figure 5). Taken together, we demonstrate that ATHB2 regulates plant development possibly through modulating the auxin pathway in *Arabidopsis*.

In common wheat, a total of 113 HD-ZIP members were identified in recent studies [38,52]. However, the relationship between wheat HD-ZIP II transcription factors and the auxin pathway remains unclear. In this study, we analyzed the canonical AuxRE distribution in the promoters of wheat HD-ZIP II subfamily genes (Figure 8). A number of AuxREs were recognized in the promoters of wheat HD-ZIP II subfamily genes. Indeed, some wheat *HD-ZIP* II subfamily genes are regulated by auxin. Such knowledge may be useful to understand the regulation of wheat HD-ZIP II transcription factor in the auxin-mediated plant developmental processes.

## 4. Methods and Materials

### 4.1. Plant Materials and Growth Conditions

All the plants described in this study were in the Col-0 background. The full-length *ATHB2* coding sequence was cloned into *pMDC7* vector (*iATHB2*) [53,54] and *p35S-FLAG* vector (*p35S:ATHB2-FLAG*) [55]*,* respectively. Col-0 was transformed with *iATHB2* and *p35S:ATHB2-FLAG* by the floral dip transformation method, respectively [56]. Two independent transgenic lines of *iATHB2* and *p35S:ATHB2-FLAG* were used for the experiments in this study. The *DR5rev:GFP/iATHB2* and *DR5:GUS/35S:ATHB2-FLAG* plants were prepared by genetic crossing. *Arabidopsis thaliana* and wheat (*Triticum aestivum*) were grown under LD (16 h light/8 h dark) condition at 22 °C.

### 4.2. DNA Constructs

DNA constructs used in this study were generated based on construction methods. The construction methods were carried out with the classic molecular biology protocols and Gateway technology (Invitrogen). For ligase-independent ligation assays, the Ligation-Free Cloning MasterMix (abm) was used according to the application handbook. For Gateway cloning, pQBV3 vector (Gateway) was used as the entry vector and subsequently specific destination vectors were introduced into the Gateway system (Invitrogen). The primers used for generation in this study are shown in Table S1.

### 4.3. Root Phenotype Analyses

Seeds were sterilized by 75% (*v*/*v*) ethanol for seven minutes and then 100% (*v*/*v*) ethanol for three minutes (*v*/*v*). Seeds were stratified at 4 °C for three days. The seeds were then grown on 1/2 MS medium with or without 10 μM β-estradiol. For the phenotypic observation, the seedlings were grown vertically on 1/2 MS containing 10 μM β-estradiol for six days. The primary root lengths were measured by using ImageJ software (http://rsb.info.nih.gov/ij).

### 4.4. RNA Extraction and Gene Expression Analysis

The 6-day-old seedlings were collected as described. Total RNA was extracted using Trizol (Invitrogen) reagent. About 2 μg total RNA was applied to synthesize cDNA using the 5× All-In-One RT MasterMix system (Applied Biological Materials). The cDNA was diluted to 100 μL with water in a 1:5 ratio, and 2 μL of the diluted cDNA was used as a template. SYBR^®^ Premix ExTaq Kit (TaKaRa) was used for qPCR reactions. qRT-PCR was performed using LightCycler 96 (Roche). Expression levels of target genes were normalized by *ACTIN7*. All the experiments were repeated independently three times. All the primers used for qRT-PCR are shown in Table S1.

### 4.5. Confocal Microscopy

Fluorescent samples were inspected by confocal microscopy (Carl Zeiss, LSM880, Germany). For imaging GFP and propidium iodide (PI) observation the 488 nm laser was used for excitation. Emission between 500 and 550 nm band-pass was detected for GFP, between 560 and 610 nm band-pass for PI.

### 4.6. Protein Extraction and Immunoblotting

The extracted buffer (125 mM Tris-HCl at pH 6.8, 4% SDS, 20% glycerol, 0.001% bromophenol blue) with freshly added 2% β-mercaptoethanol was used for total protein extraction. Immunoblots were performed as described [57]. To detect a FLAG-tagged protein, we used anti-FLAG (1:5000; MBL, Japan) and anti-mouse IgG (1:75000) antibodies. Actins (1:5000; CWBIO) were used as the loading controls. Three independent biological replicates were performed with similar results.

### 4.7. Analysis of GUS Activity

For GUS activity analysis, 4-week-old *Arabidopsis* leaves were transferred into staining solution (1 mM 5-bromo-4-chloro-3-indolyl-beta-glucuronic acid solution in 100 mM sodium phosphate, pH 7.0, 0.1 mM EDTA, 0.5 mM ferricyanide, 0.5 mM ferrocyanide and 0.1% Triton X-100) [58]. Leaves were then applied to vacuum for 20 min and incubated at 37 °C overnight. To clear chlorophyll from plant tissues, 100% ethanol was used. Individual representative seedlings were photographed.

### 4.8. Phylogenetic Analysis

A neighbor-joining phylogenetic tree was constructed based on 1000 bootstrap replicates by comparing full-length protein sequences aligned with the Clustal W algorithm within MEGA7.0.

### 4.9. Protein Structure and Motif Composition Analyses

Predicted protein domains were identified by the SMART tool (http://smart.emblheidelberg.de/). The MEME online program (Bailey et al., 2009) was used to identify conserved motifs.

### 4.10. Accession Numbers

Sequence data of *Arabidopsis* from this article can be found in the *Arabidopsis* Genome initiative data library under the following accession numbers: *ATHB2* (AT4G16780), *IAA19* (AT3G15540), *YUC2* (AT4G13260), *YUC8* (AT4G28720), *PIN1* (AT1G73590), *PIN2* (AT5G57090), *PIN3* (AT1G70940), *PIN4* (AT2G01420), *ATHB4* (AT2G44910), *ATHB17* (AT2G01430), *ATHB18* (AT1G70920), *HAT1* (AT4G17460), *HAT2* (AT5G47370), *HAT9* (AT2G22800), *HAT14* (AT5G06710) and *HAT22* (AT4G37790).

## Supplementary Materials

Supplementary materials can be found at https://www.mdpi.com/1422-0067/21/9/3250/s1.
