# Supplementary material for: The HD-ZIP II Transcription Factors Regulate Plant Architecture through the Auxin Pathway
Source: Int J Mol Sci. 2020 May 4;21(9):3250. doi: 10.3390/ijms21093250 (PMC7246542; doi:10.3390/ijms21093250)
Supplement: Supplementary file 1 [file ijms-21-03250-s001.pdf]

Supplemetal Figure 1

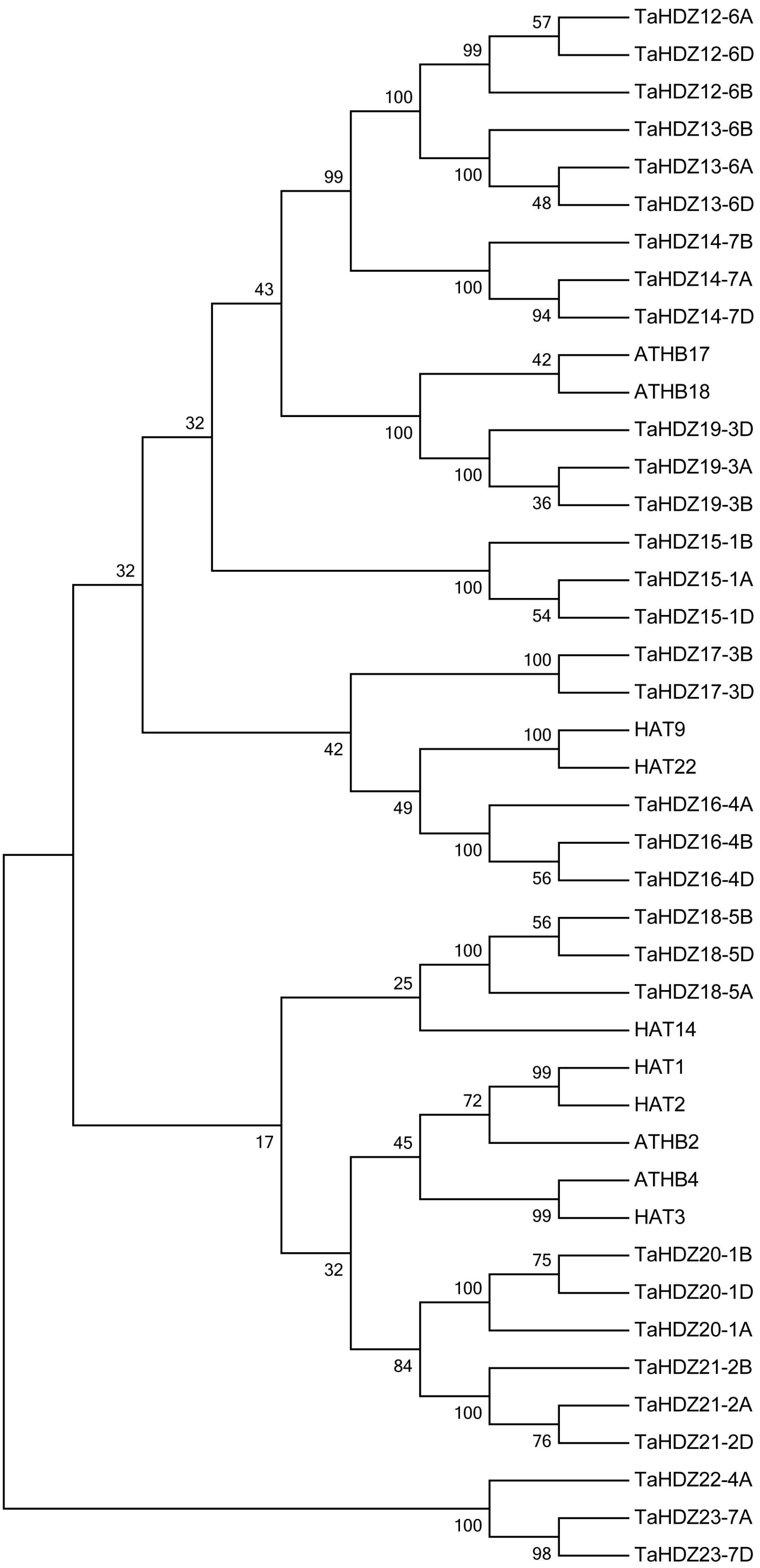

Supplemental Figure 1. Phylogenetic Analyses of the HD-ZIP II Subfamily Proteins from *Arabidopsis* and Wheat. The phylogenetic tree was constructed via MEGA7.0 software.

Supplemetal Figure 2

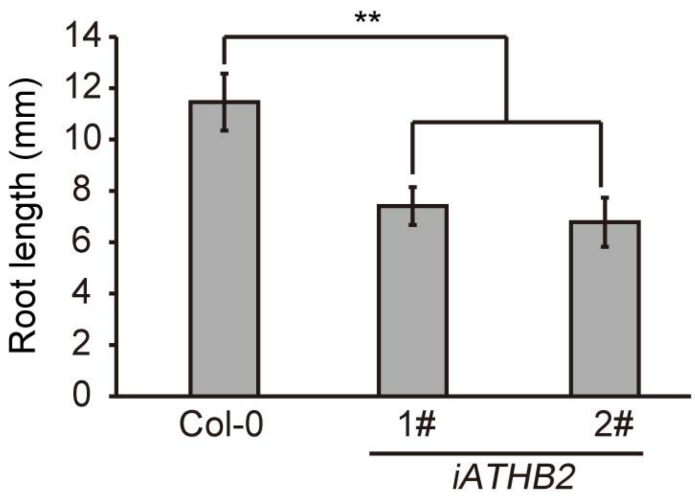

Supplemental Figure 2. The Primary Root Length of Col-0 and *iATHB2* transgenic Seedlings. The seedlings of Col-0 and inducible-*ATHB2* plants (*iATHB2*) were grown on 1/2 MS medium with 10 μM β-estradiol for 5.5 days. Two independent transgenic lines were used for root length measurements. The data are representative of three independent experiments.

Supplemetal Figure 3

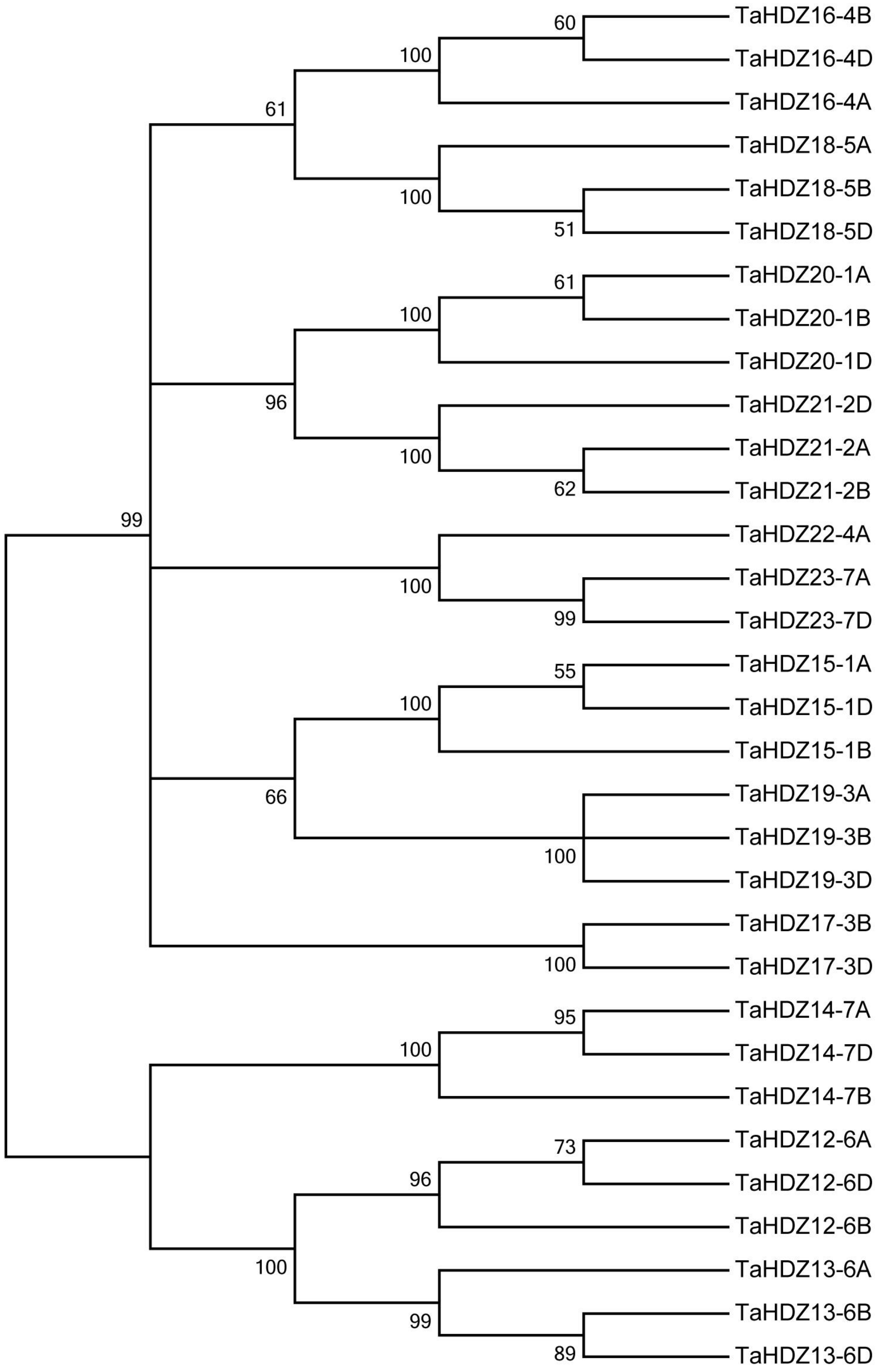

Supplemental Figure 3. Phylogenetic Analyses of the HD-ZIP II Subfamily Proteins in Wheat. The phylogenetic tree was constructed via MEGA7.0 software.

Supplemetal Figure 4

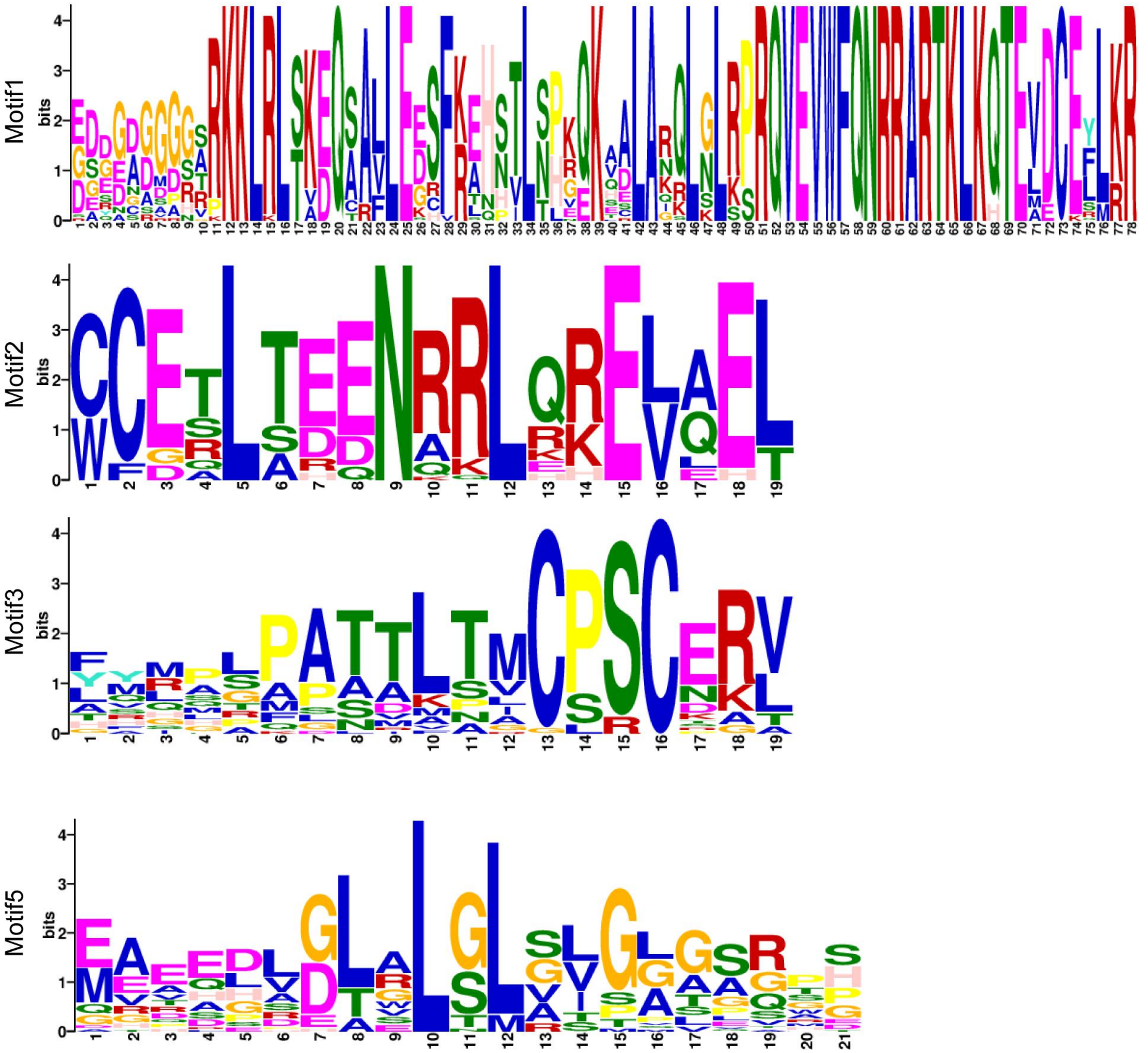

Supplemental Figure 4. Logos for predicted conserved domains found in 32 wheat HD-ZIP II subfamily proteins by MEME program.

Supplemental Table 1. Constructs and Primers Used in This Study.

Assays	Destination products	Templet	Primer name	Primer sequence (5'-3')
qRT-PCR		ATHB2	ATHB2-F	AAATCCATCTGTTTCTGTTACTCCTT
			ATHB2-R	TGTGACGAATCTGAGTTTGGA
		HAT1	HAT1-F	AACAAAAGCAACAGTTTCTTAGG
			HAT1-R	AAGATCTATCTAGAGTGATGTCAAGG
		HAT2	HAT2-F	ATCCTACATCAGATCTTCGCAAG
			HAT2-R	AGTACCCTCGATCCGGAGTG
		HAT3	HAT3-F	TTCTTATGCATCTTCATCACACATG
			HAT3-R	TCTATTCCCCGGAGAAACG
		ATHB4	ATHB4-F	TGGGATTAAACGTACAACTGC
			ATHB4-R	CGCAAGATCCCTTTTATTTC
		IAA19	IAA19-F	TGGTTCGAGCCAAGGCTATGATG
			IAA19-R	CATCTTTCAAGGCCACACCGATGC
		ACTIN7	ACTIN7-F	TCCATGAAACAACTTACAACTCCATCA
			ACTIN7-R	CATCGTACTCACTCTTTGAAATCCACA
		YUC2	YUC2-F	GGTGACACGGATCGGTTAGGGT
			YUC2-R	TGCCGAATAATGCATTACCCGT
		YUC8	YUC8-F	TGTATGCGGTTGGGTTTACGAGGA
			YUC8-R	CCTTGAGCGTTTCGTGGGTTGTTT
		PIN1	PIN1-F	GGTCGGAACTCTAACTTTGGTC
			PIN1-R	CAGCTCCAGCAGCAGTTCCAGC
		PIN2	PIN2-F	CCTCGCCGCACTCTTTCTTTGG
			PIN2-R	CCGTACATCGCCCTAAGCAATGG
		PIN3	PIN3-F	AGCACCTGACAACGATCAAGGCG
			PIN3-R	GTTCTCCTCCGAAATCTCCACT
		PIN4	PIN4-F	TCTTGAGACGGATGCTGAGATA
			PIN4-R	TCAGCTCCGGTAAGATTCGA
		TaGAPDH	TaGAPDH -F	TTAGACTTGCGAAGCCAGCA
			TaGAPDH -R	AAATGCCCTTGAGGTTTCCC
		TaHDZ19	TaHDZ19-F	GGCTCAGAGGGGTTTGTTTG
			TaHDZ19-R	TCCTCCTCGTCCTCTTCCA
		TaHDZ20	TaHDZ20-F	TGATTGTTAACGGGAGGGACA
			TaHDZ20-R	AGGAGGCCGGATAGAAGAGG
		TaHDZ21	TaHDZ21-F	ATGGAGAGGGCCGAGGAC
			TaHDZ21-R	GGCATCTCCAGTAATCTCCTCTC
		TaHDZ23	TaHDZ23-F	TTTCCCAGCCCACCCTG
			TaHDZ23-R	AGCACCTCCTCTCCTCCACC
Transformation	iATHB2	ATHB2	ATHB2-F1	ATGATGTTCGAGAAAGACGATCT
			ATHB2-R1	GGACCTAGGACGAAGAGCGTC
	35S:ATHB2-Flag	ATHB2	ATHB2-F2	CTCGAGGCGCGCCGTCGACGATGATGTTCGAGAAAGACGATCT
			ATHB2-R2	CCTTGTAGTCCATGTCGACGGACCTAGGACGAAGAGCGTC

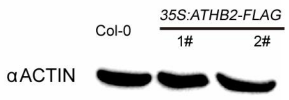

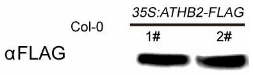

The uncropped, untouched, full original images of western blots of Figure 2A.
